# A Mirror-Image Heartache: Reflections on a False Diagnosis of Acute Coronary Syndrome in a Patient With Situs Inversus Totalis

**DOI:** 10.7759/cureus.97143

**Published:** 2025-11-18

**Authors:** Armin B Bassi, Abdulrazak A Mohamad

**Affiliations:** 1 General Medicine, King's College Hospital NHS Foundation Trust, London, GBR; 2 Internal and Interventional Medicine, Hunter New England Health, Newcastle, AUS

**Keywords:** acute coronary syndrome (acs), chest imaging, chest pain, diagnostic error, electrocardiography (ecg), situs-inversus-totalis

## Abstract

*Situs inversus totalis* (SIT) is a rare congenital anomaly characterized by complete mirror-image transposition of the thoracic and abdominal organs. Unrecognized SIT can create diagnostic confusion in patients presenting with chest pain, as standard electrocardiographic (ECG) lead placement may generate waveforms that mimic acute coronary syndromes (ACS). We report the case of a 30-year-old physically fit man who presented with acute central chest pain following exertion. Initial investigations revealed nonspecific ECG abnormalities and a mildly elevated high-sensitivity troponin level, leading to a presumptive working diagnosis of ACS. On structured re-examination, a right-sided apex beat was identified, and chest radiography confirmed dextrocardia. A repeat ECG performed with mirror-image precordial lead placement restored normal waveforms, and serial cardiac biomarkers showed no dynamic trend, confirming the absence of myocardial infarction. This case illustrates that even in modern, protocol-driven medicine, diagnostic precision in acute chest-pain evaluation depends on clinical judgment derived from correlating systematic bedside assessment with multimodal investigation, including imaging, ECG, and biomarker analysis. This integrative approach enhances diagnostic accuracy, prevents unnecessary interventions, and promotes safe, patient-centered outcomes, particularly in individuals with rare anatomic variants such as SIT.

## Introduction

Situs inversus totalis (SIT) is a rare congenital condition characterized by complete mirror-image transposition of the thoracic and abdominal organs, with an estimated incidence of approximately 1 in 10,000 to 20,000 live births [1]. This anatomical reversal arises from disruption of left-right axis determination during early embryogenesis, driven by aberrations in ciliary motion and signaling pathways such as NODAL, LEFTY, and PITX2, which normally establish visceral laterality [1]. While SIT itself is typically benign, its presence can have important implications for diagnostic interpretation, procedural planning, and emergency management [2,3].

SIT may occur in isolation or, more rarely, as part of Kartagener syndrome, where it coexists with chronic sinusitis and bronchiectasis secondary to primary ciliary dyskinesia (PCD). In syndromic forms, symptoms often manifest in childhood with recurrent respiratory infections, sinusitis, or pneumonia resulting from impaired mucociliary clearance [4]. In contrast, isolated SIT is entirely asymptomatic and may remain undetected throughout life, often discovered incidentally during imaging, surgery, or unrelated clinical evaluations [3]. When unrecognized, mirror-image anatomy can complicate clinical evaluation and create diagnostic confusion across medical and surgical specialties [2,3]. This is particularly important in acute presentations such as chest pain, where unrecognized dextrocardia may lead to misinterpretation of electrocardiographic (ECG) findings, as standard lead placement can generate waveforms that mimic acute coronary syndromes (ACS) [5,6].

According to current definitions, ACS encompasses a spectrum ranging from unstable angina to ST-elevation and non-ST-elevation myocardial infarction, each diagnosed through a combination of characteristic symptoms, ECG changes, and a dynamic rise or fall in cardiac biomarkers [7,8]. In patients with SIT, these diagnostic pillars can be misleading: mirror-image cardiac anatomy may distort waveform morphology, risking inappropriate activation of ACS pathways, unnecessary invasive procedures, and delayed recognition of the true underlying cause [9,10].

We describe a young patient with previously undiagnosed SIT who presented with acute chest pain and was initially managed under a working diagnosis of ACS, which was subsequently revised following re-evaluation. This case exemplifies how diagnostic precision in acute chest-pain evaluation depends on correlating and contextualizing thorough bedside assessment with imaging, ECG, and biochemical investigations. Integrating these findings into clinical reasoning enables clinicians to recognize and avoid diagnostic pitfalls in complex presentations, such as unrecognized anatomical variants, ensuring safe, patient-centered care.

## Case presentation

History

A 30-year-old physically fit man presented with a five-hour history of sudden-onset, continuous central chest pain that began during a vigorous gym workout. The pain was sharp, non-radiating, and exacerbated by deep inspiration and coughing. He rated the pain as 6/10 in intensity and denied diaphoresis, clamminess, dyspnea, nausea, or palpitations.

The patient's past medical history was unremarkable, and he was not taking any regular medications. He reported a two-year history of smoking (half a pack per day) and moderate alcohol intake but denied illicit drug use. His family history was notable for myocardial infarction affecting both his father and paternal grandfather in their 60s.

Observations and examination

On presentation, vital signs were within normal limits: temperature 36.8 °C, heart rate 86 bpm, blood pressure 124/78 mmHg, respiratory rate 16/min, and oxygen saturation 99% on room air. Cardiac auscultation revealed normal S₁ and S₂ at conventional precordial sites. No murmurs, rubs, or additional heart sounds were appreciated.

Lung fields were clear bilaterally, and no added sounds were detected. Palpation of the costochondral joints reproduced the patient's chest pain, suggesting a possible musculoskeletal component. The abdomen was soft, non-tender, and without palpable organomegaly. Peripheral examination revealed no edema, cyanosis, or clubbing.

Biochemistry

Initial laboratory investigations (Table 1) demonstrated normal complete blood count, renal function, electrolytes, liver function tests, and inflammatory markers. D-dimer was within normal limits. High-sensitivity troponin I was mildly elevated at 54 ng/L (reference < 14 ng/L).

**Table 1:** Summary of Laboratory Investigations Initial laboratory investigations on arrival at the emergency department. Values were within normal limits except for a mildly elevated high-sensitivity troponin I.

Parameter	Patient Result	Reference Range
Hemoglobin	14.8 g/dL	13.0–17.0 g/dL
White Blood Cell Count	6.5 × 10⁹/L	4.0–11.0 × 10⁹/L
Platelet Count	250 × 10⁹/L	150–400 × 10⁹/L
Mean Corpuscular Volume (MCV)	88 fL	80–100 fL
Sodium	139 mmol/L	135–145 mmol/L
Potassium	4.2 mmol/L	3.5–5.0 mmol/L
Urea (BUN)	5.0 mmol/L	2.5–7.5 mmol/L
Creatinine	80 µmol/L	60–110 µmol/L
Estimated Glomerular Filtration Rate (eGFR)	> 90 mL/min/1.73 m²	> 60 mL/min/1.73 m²
Alanine Aminotransferase (ALT)	25 U/L	< 45 U/L
Aspartate Aminotransferase (AST)	22 U/L	< 40 U/L
Alkaline Phosphatase (ALP)	90 U/L	40–130 U/L
Total Bilirubin	0.8 mg/dL (14 µmol/L)	< 1.2 mg/dL (< 20 µmol/L)
Albumin	42 g/L	35–50 g/L
C-Reactive Protein (CRP)	3 mg/L	< 5 mg/L
High-Sensitivity Troponin I	54 ng/L	< 14 ng/L

Electrocardiography

**Figure 1:**
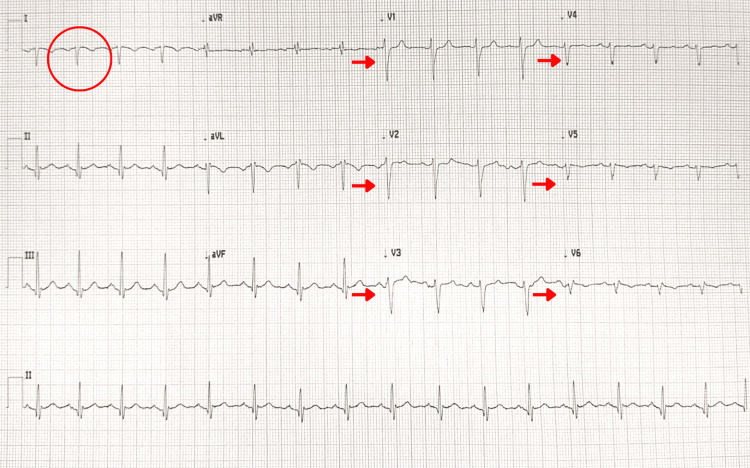
Electrocardiogram With Standard Left-Sided Precordial Lead Placement The ECG demonstrates sinus rhythm with right-axis deviation, global QRS negativity, and inverted P and T waves in lead I (circle). There is progressively decreasing R-wave amplitude from leads V1 to V6 (arrows), consistent with poor R-wave progression.

A standard 12-lead ECG demonstrated sinus rhythm with right-axis deviation, global QRS negativity, and inverted P and T waves in leads I and aVL. Poor R-wave progression was observed across the precordial leads (Figure 1).

These findings, in conjunction with the patient's exertional central chest pain and elevated high-sensitivity troponin level, raised concern for an ACS and fulfilled preliminary elements of the Fourth Universal Definition of Myocardial Infarction, pending confirmation by serial biomarker testing [7]. Activation of the hospital's ACS pathway was undertaken in accordance with local emergency protocols, and the patient was commenced on dual antiplatelet therapy (DAPT) and sublingual glyceryl trinitrate (GTN) while awaiting urgent cardiology review. Given the atypical features of the pain and the patient's stable condition, the initial differential diagnoses included early ACS, myopericarditis, and musculoskeletal chest pain.

Re-evaluation

Given the patient's stable condition, the specialty team undertook targeted re-evaluation before proceeding with invasive testing. A focused cardiovascular examination identified the apex beat in the right fifth intercostal space. A bedside transthoracic ECG demonstrated normal biventricular function with no regional wall-motion abnormalities. A mirror-image atrial arrangement was observed, and the cardiac apex was directed rightward. As this was a point-of-care assessment, images were not stored for publication. The absence of pericardial rub, PR-segment depression, or diffuse ST-segment elevation on ECG, together with normal inflammatory markers and echocardiographic findings, made myopericarditis unlikely.

**Figure 2:**
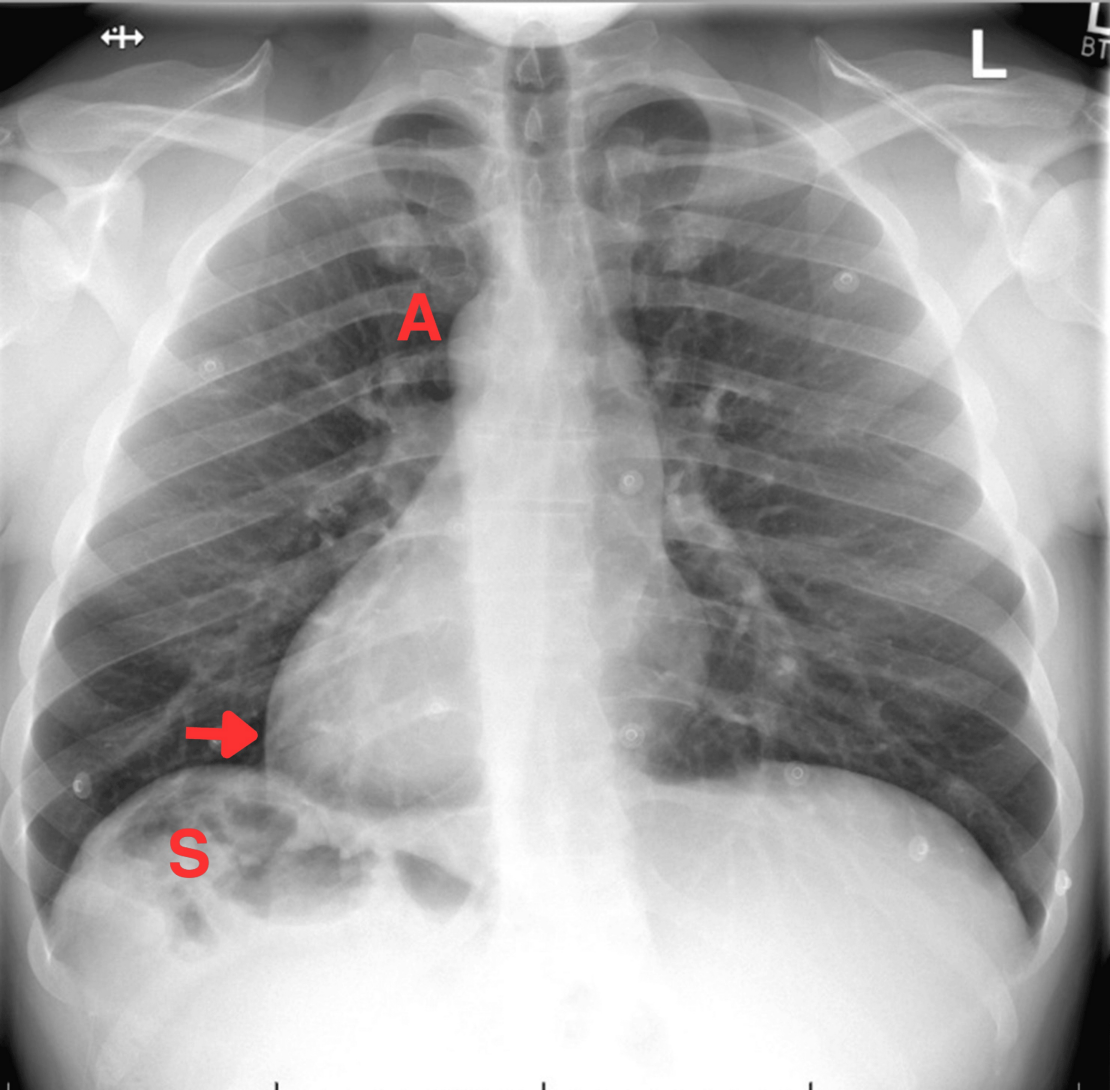
Chest Radiograph Demonstrating Situs Inversus Totalis An anteroposterior (AP) chest radiograph demonstrating dextrocardia, with the cardiac apex (arrow) and aortic arch (A) positioned on the right side of the thorax. A gastric air bubble (S) is visible beneath the right hemidiaphragm, confirming right-sided stomach orientation. These findings are diagnostic of situs inversus totalis (SIT).

**Figure 3:**
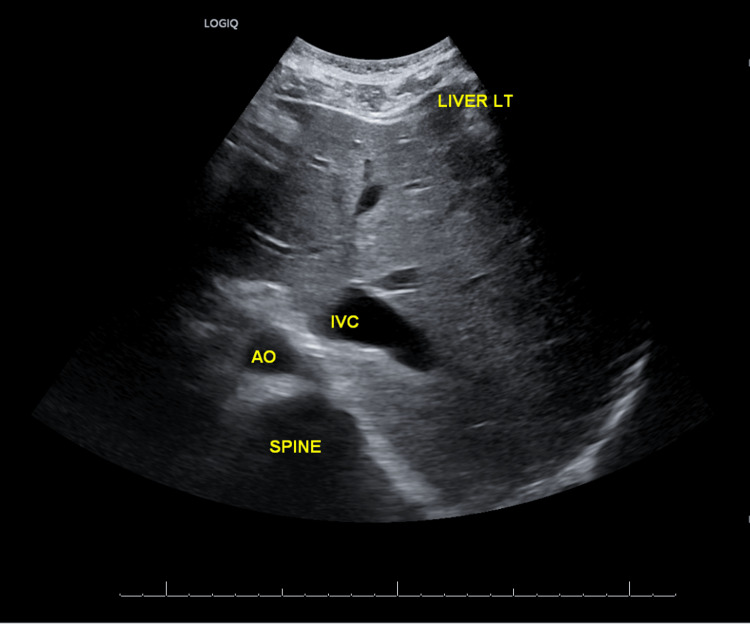
Abdominal Ultrasound Demonstrating Mirror-Image Organ Orientation in Situs Inversus Totalis Subcostal ultrasound view demonstrating the liver (LIVER) and inferior vena cava (IVC) positioned on the left of midline and the aorta (A) on the right, confirming mirror-image abdominal organ orientation consistent with situs inversus totalis (SIT). The spleen was also visualized in the right upper quadrant during scanning but is not included in this image.

These unexpected findings prompted consideration of dextrocardia and guided the decision to obtain confirmatory imaging. Chest radiography revealed dextrocardia (Figure 2), while abdominal ultrasound demonstrated complete mirror-image organ orientation, with the liver and inferior vena cava (IVC) positioned on the left and the aorta on the right (Figure 3). The spleen was visualized in the right upper abdomen during scanning, though not captured in the published image.

**Figure 4:**
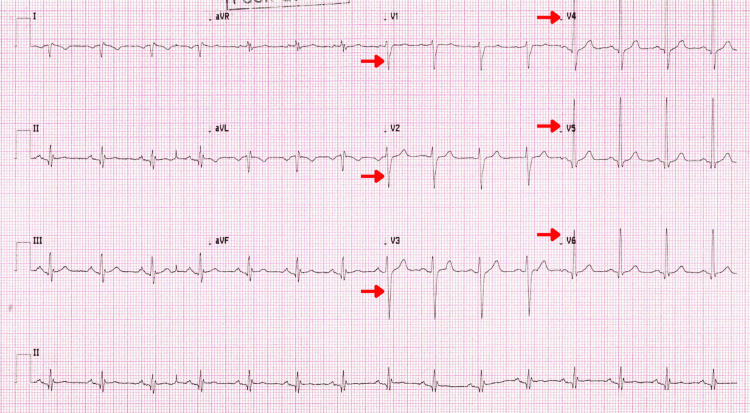
Electrocardiogram With Mirror-Image (Right-Sided) Precordial Lead Placement Repeat 12-lead electrocardiogram performed with mirror-image (right-sided) precordial lead placement, in which leads V1–V6 were positioned on the corresponding intercostal spaces of the right hemithorax while limb leads remained in standard configuration. The tracing demonstrates normalization of waveforms, with restored R-wave progression across leads V1–V6 (arrows), confirming correct right-sided cardiac orientation in situs inversus totalis (SIT).

A repeat ECG was performed with mirror-image (right-sided) precordial lead placement, in which leads V1-V6 were positioned over the corresponding intercostal spaces of the right hemithorax while the limb leads remained in standard configuration. This adjustment demonstrated normalization of waveforms, with restoration of R-wave progression across the precordial leads (Figure 4). Serial ECGs and repeat high-sensitivity troponin testing performed three hours later showed no dynamic change (51 ng/L compared to 54 ng/L at baseline), confirming the absence of myocardial infarction. Furthermore, the absence of pericardial rub, PR-segment depression, or diffuse ST-segment elevation on ECG, together with normal inflammatory markers and echocardiographic findings, made myopericarditis unlikely.

Outcome and follow-up

The absence of dynamic troponin change, normalization of the ECG after right-sided precordial lead placement, and concordant imaging findings confirmed that the presentation was non-ischemic in origin. DAPT and GTN were discontinued, and the patient was reassured regarding the benign nature of his anatomical variant. He was discharged with nonsteroidal anti-inflammatory drugs (NSAIDs) for symptomatic relief of chest wall discomfort, consistent with a musculoskeletal etiology.

Prior to discharge, the patient received education regarding his diagnosis of SIT. He was advised to inform healthcare providers of his condition in future presentations, as the mirror-image organ orientation may alter the interpretation of ECGs, imaging, and procedural approaches. A medical alert bracelet was recommended for this purpose.

Although he denied chronic cough, sinus symptoms, or recurrent respiratory infections, his primary care provider was advised to monitor for respiratory manifestations suggestive of PCD. The primary care provider was also advised to arrange a baseline echocardiographic study to exclude structural cardiac anomalies and to reinforce smoking cessation and cardiovascular risk reduction, given the positive family history of myocardial infarction.

At review in the Rapid Access Chest Pain Clinic three days later, the patient reported complete resolution of symptoms. Repeat high-sensitivity troponin levels remained unchanged, and exercise treadmill testing was normal. No further cardiology follow-up was required, and he was discharged to community care.

Written informed consent was obtained from the patient for publication of this case report and accompanying images. The patient was informed that no identifying information would be disclosed, and every effort has been made to ensure anonymity.

## Discussion

SIT is a rare congenital condition seldom encountered in routine clinical practice. Although most individuals remain asymptomatic, undetected cases can present significant diagnostic challenges, particularly in acute settings where standardized, protocol-driven approaches dominate clinical decision-making [2,3,11]. In the present case, the combination of exertional chest pain, abnormal ECG findings, and a mildly elevated high-sensitivity troponin level prompted activation of the ACS pathway. While the initial management appropriately followed established ACS protocols [8], the patient's mirror-image cardiac orientation produced artifactual ECG changes that mimicked myocardial ischemia, leading to an initial misclassification that was subsequently clarified through clinical re-evaluation.

ECG misinterpretation is well documented in dextrocardia and SIT [5,6]. In these patients, standard left-sided precordial lead placement reverses the spatial relationship between the heart and electrodes, producing apparent right-axis deviation, global QRS negativity, and inverted P and T waves in lead I, along with reversed or absent R-wave progression in the precordial leads. A positive aVR accentuates the abnormal frontal-plane axis and should raise suspicion for dextrocardia when seen alongside these features [12,13]. Recognition of these distinctive patterns enables clinicians to differentiate artifactual changes from true ACS phenomena, preventing unnecessary interventions and delays in management.

The diagnostic uncertainty in this case was compounded by a mildly elevated high-sensitivity troponin I level (54 ng/L), which met the biochemical threshold for myocardial injury [7]. However, the absence of a dynamic rise or fall, combined with recent strenuous exercise, suggested exercise-induced myocyte leak rather than true ischemia [14,15]. This highlights the importance of correlating biochemical data with clinical and imaging findings, as troponin elevations can occur in a range of non-ischemic contexts, including strenuous exertion, tachyarrhythmia, myocarditis, or systemic illness [16].

Reversing the precordial ECG leads to a mirrored configuration that realigns the recording vectors with the heart's true anatomic orientation, thereby normalizing aberrant waveforms, restoring R-wave progression, and correcting inverted complexes typical of SIT [5,12]. In our case, only the precordial leads were reversed, which corrected the poor R-wave progression while maintaining the limb leads in their standard configuration. This selective reversal enabled accurate assessment of the right-sided cardiac position without distorting the frontal-plane axis. By analyzing the ECG from two perspectives, the adjusted precordial vectors reflecting the true anatomic orientation and the unchanged limb leads preserving the standard reference, our team was able to distinguish static anatomic artifacts from true dynamic ischemic changes [5,9,13].

The diagnostic turning point occurred during a focused cardiovascular re-examination, when the apex beat was identified in the right fifth intercostal space. This observation, initially overlooked during standard left-sided assessment, prompted reconsideration of cardiac orientation and guided subsequent imaging. Chest radiography confirmed dextrocardia, while abdominal ultrasound demonstrated complete visceral transposition, establishing the diagnosis of SIT and excluding heterotaxy or situs ambiguus [17,18]. This sequence highlights the enduring centrality of clinical acumen: even within protocol-driven environments, bedside examination remains the foundation of diagnostic accuracy. In this case, imaging worked in tandem with clinical findings to confirm and contextualize the diagnosis.

International guidelines for the evaluation of acute chest pain recognize chest radiography as a valuable adjunct for assessing non-ischemic thoracic causes, such as pulmonary, pleural, or aortic pathology, and endorse its early use in appropriate cases [8]. This case underscores the complementary role of early chest radiography in acute chest-pain evaluation. In hemodynamically stable patients, a simple radiograph can rapidly orient clinicians to the cardiac apex and mediastinal position, offering immediate visual confirmation of anatomic variants that may otherwise confound ECG interpretation and procedural planning. By contextualizing ECG and biomarker findings within this anatomical framework, clinicians can achieve greater diagnostic precision and avoid unnecessary escalation of care. Nevertheless, radiography should always be applied judiciously and never at the expense of timely reperfusion therapy in critically unwell patients with clear evidence of acute coronary occlusion.

Ultimately, this case illustrates how protocol-based pathways and individualized clinical reasoning can operate synergistically rather than in opposition. Adherence to established ACS pathways provided a safe framework for initial management, while iterative bedside assessment and multimodal evaluation refined the diagnosis with accuracy and precision. It further reinforces that clinical examination remains the cornerstone of modern diagnostic reasoning and that technology should augment, rather than replace, clinical insight. Although this report details a single case, it exemplifies broader diagnostic principles essential to the assessment of complex chest-pain presentations in acute care, particularly when rare anatomical variants may obscure the underlying diagnosis.

## Conclusions

This case underscores the importance of a multimodal diagnostic approach when evaluating patients presenting with acute chest pain. In our patient with previously unrecognized SIT, the rediscovery of a right-sided apex beat during re-examination was the decisive turning point that guided appropriate imaging, clarified ECG interpretation, and prevented unnecessary intervention. The sequence underscores that even within highly standardized pathways such as those for ACS, diagnostic precision depends on correlating physical findings with imaging, ECG, and biomarker trends. Imaging investigations served as pivotal adjuncts, confirming the mirror-image anatomy and contextualizing the biochemical and ECG data. Ultimately, this case reaffirms that in modern, protocol-driven medicine, the most reliable safeguard against diagnostic error remains the thoughtful fusion of clinical acumen with evidence-based investigation.
